# Expression profiles of circRNAs and the potential diagnostic value of serum circMARK3 in human acute Stanford type A aortic dissection

**DOI:** 10.1371/journal.pone.0219013

**Published:** 2019-06-28

**Authors:** Congcong Tian, Xinlong Tang, Xiyu Zhu, Qing Zhou, Yuting Guo, Rui Zhao, Dongjin Wang, Bing Gong

**Affiliations:** 1 Department of Cardiothoracic Surgery, Nanjing University Medical School Affiliated Drum Tower Hospital, Nanjing, China; 2 National Laboratory of Biomacromolecules, CAS Center for Excellence in Biomacromolecules, Institute of Biophysics, Chinese Academy of Sciences, Beijing, China; 3 College of Life Sciences, University of Chinese Academy of Sciences, Beijing, China; 4 State Key Laboratory of Cardiovascular Disease, Fuwai Hospital, National Center for Cardiovascular Diseases, Chinese Academy of Medical Sciences and Peking Union Medical College, Beijing, People's Republic of China; Thomas Jefferson University, UNITED STATES

## Abstract

CircRNAs are involved in a variety of human diseases, however, the expression profiles and the potential diagnostic value of circRNAs in human acute Stanford type A aortic dissection (AAAD) remains largely unknown. In this study, high-throughput RNA sequencing (RNA-Seq) was used to investigate the differentially expressed circRNAs, microRNAs (miRs) and mRNAs in human AAAD tissues (n = 10) compared with normal aortic tissues (n = 10). The results of RNA-Seq revealed that 506 circRNAs were significantly dysregulated (*P*<0.05, false discovery rate, FDR<0.05, fold change>2). The subsequent weighted gene correlation network analysis and the following co-expression network analysis revealed that tyrosine-protein kinase Fgr might play important roles in the occurrence and development of AAAD. According to the circRNA-miRNA-mRNA network, we found that the upstream regulatory molecule of Fgr is circMARK3. Finally, a receiver operating characteristic (ROC) curve was used to evaluate the diagnostic value of the serum circMARK3 as biomarkers for AAAD (cutoff value = 1.497, area under the curve = 0.9344, *P* < 0.0001, sensitivity = 90.0%, specificity = 86.7%). These results provided a preliminary landscape of circRNAs expression profiles and indicated that circMARK3 was a potential biomarker for AAAD diagnosis.

## Introduction

Acute Stanford type A aortic dissection (AAAD) is one of the most dangerous vascular diseases, which is characterized by the tear of the aortic wall. Once the AAAD occurs, the mortality rate is extremely high, related epidemiological studies showed that the mortality of untreated patients with AAAD is 50% (36–72%) within 48 hours [1, 2]. Although various treatments for AAAD have been significantly improved, the incidence of complications and mortality of patients with AAAD are still very high [3]. The etiology of AAAD is diverse and influenced by genetic components and hemodynamic stress [4, 5]. AAAD is commonly associated with aortic medial degeneration [6]. Although the main mechanisms that contribute to aortic medial degeneration are extracellular matrix degradation [7], apoptosis and phenotype switch of smooth muscle cells [8] and inflammation [9], the precise trigger of aortic dissection is unknown [10].

Besides important functional proteins, more and more studies have revealed that non-coding RNAs also play vital roles in the progression of many kinds of aortic diseases [11, 12]. MicroRNAs (miRs) and long non-coding RNAs (lncRNAs) are two well studied non-coding RNAs, and some studies have provided evidence that certain miRs and lncRNAs are involved in the molecular mechanism of AAAD [11–13]. For example, miR-21 has been considered to be a regulator of thoracic aortic dissection in mice through transforming growth factor-β-SMAD3 signaling [13], and lncRNA XIST might play important roles in AAAD by sponging miR-17-5p and regulating p21 [12]. Owing to the development of high-throughput RNA sequencing (RNA-Seq) technology, increasing variety of non-coding RNAs such as circular RNAs (circRNAs) have been found [14]. Unlike miRs and lncRNAs, circRNAs are covalently closed looped circular RNAs, which are characterized by extraordinary stability [15]. Related studies have suggested circRNAs play vital roles in the initiation and progression of a number of human diseases [16–18]. However, little is known about the landscape of circRNAs profiles and their diagnostic value in AAAD.

CircRNAs were reported to have the ability to act as the miRNA sponges, which inhibit miRs access to their target mRNAs by competing for the same binding site of miRs, thereby regulating the target gene of the respective miRs [16]. Constructing a circRNA-miRNA-mRNA network could make us have a better understanding of the biofunction of the differentially expressed circRNAs in AAAD.

In this study, we screened the circRNAs expression profiles of AAAD using RNA-Seq assay. Bioinformatic analysis was performed to predict the potential biofunction of the differentially expressed circRNAs (AAAD vs control). Tyrosine-protein kinase Fgr, which participates in transmitting extracellular signals into cells upon extracellular stimulation [19], regulating immune response [20, 21] and the release of inflammatory factors, was hypothesized to play an important role in the development of AAAD and its upstream regulator, serum circMARK3, was hypothesized to have potential diagnosis value.

## Materials and methods

### Patients and specimens

This study was conducted in accordance with the Declaration of Helsinki and was approved by the Medical Ethics Committee of Nanjing Drum Tower Hospital, the affiliated hospital of Nanjing university medical school (Institutional Review Board File 2016-152-01). Subjects (or their guardians) have given their written informed consent. Diseased ascending aortic specimens were obtained from AAAD patients undergoing aortic replacement surgery in our center. All the AAAD patients were verified using contrast-enhanced CT. The normal ascending aortic wall specimens were obtained from patients undergoing coronary artery bypass grafting surgery (CABG) without aortic disease. During the study period, 67 AAAD patients were admitted to our center, 8 patients were excluded (2 patients refused to participate and 6 patients were diagnosed as Marfan syndrome). And 176 CABG patients were admitted to our center, 4 patients who refused to participate were excluded. At last, 59 AAAD patients and 172 CABG patients were enrolled in this study.

Propensity score matching (PSM) was performed to reduce bias in this study. A multivariate logistic regression model was used to calculate the propensity score for each patient. The covariables used to build the propensity score were age, gender, body mass index, smoking, hypertension, and diabetes mellitus. The CABG patients were matched to AAAD patients using a 1:1 nearest neighbor matching algorithm without replacement (caliper 0.05), resulting in cohorts that were well balanced on baseline characteristics. PSM was performed using SPSS 22 (IBM software; version 22). Based on this propensity score matching, 30 AAAD patients and 30 controls were included in this study. For the RNA-Seq discovery cohort, a subset comprising of 10 AAAD samples and 10 matched controls was used. All 30 matched specimens from the two groups were used in the following biological experiments.

### RNA preparation for RNA-Seq

Total RNA was extracted from each sample using TRIzol Reagent (Life technologies) according to the protocol from manufacturer. The concentration of each sample was measured by NanoDrop 2000 (Thermo Scientific). The quality was assessed by the Agilent2200 (Agilent) (S3 Table). The RNA integrity was assessed by electrophoresis with denaturing agarose gel.

### cDNA library preparation, microRNA library preparation and sequencing

Total RNA from each sample was used to construct the cDNA libraries using the VAHTSTM Total RNA-Seq (H/M/R). The microRNA libraries were constructed using Ion Total RNA-Seq Kit v2.0. The protocols have been described in detail in a previous publication [22]. RNA-Seq was performed by the laboratory of Shanghai NovelBio Company.

### Identification of differentially expressed circRNAs, miRs and mRNAs

The DE-Seq algorithm was applied to filter differentially expressed circRNAs, miRs and mRNAs. Based on fold change (either <0.5 or >2.0), significance analysis (*P*<0.05), and false discovery rate (FDR; *P* <0.05) analysis, differentially expressed circRNAs, miRs, and mRNAs were selected for further exploration in the full sample sets.

### Bioinformatics analysis

The differentially expressed (AAAD vs control) circRNAs, miRs and mRNAs were analyzed together to construct the circRNA-miRNA-mRNA interaction network. The base-pairing and the minimum free energy (mfe) for the binding of miRs to its targeting sequences were calculated by the RNAhybrid program. The co-expression network was drawn with the Cytoscape software (version 3.6.1).

Gene Ontology (GO) analysis and Kyoto Encyclopedia of Genes and Genomes (KEGG) pathway analysis were performed for the target genes of differentially expressed circRNAs using clusterProfiler package (version 3.8.1) within Bioconductor project (version 3.7) in R software (version 3.5.1). The enrichment thresholds were *P*<0.05 and the gene counts≥2.

Gene set enrichment analysis (GSEA) was conducted and the plots were generated by GSEA (version 3.0). It was used to identify up- or down-regulation of functionally related groups of genes (gene sets) with statistically significant enrichment. The gene set databases of c5.all.v6.2.symbols.gmt [Gene ontology] and c2.cp.kegg.v6.2.symbols.gmt [Curated] were used. GSEA performed 1000 permutation. Collapse dataset to gene symbols was false and permutation type was gene_set. Enrichment statistic was weighted. The metric used for ranking genes was Signal2Noise. The gene list sorting mode was real and displayed in descending ordering. The size for gene sets was between 15 and 500.

Weighted gene co-expression network analysis (WGCNA) was performed using R (version 3.5.1) by the WGCNA package (version 1.67) [23]. The target genes of differentially expressed circRNAs were input to WGCNA. The soft thresholding power for constructing the WGCNA adjacency matrix was calculated to be 16 using the pickSoftThreshold function in the WGCNA R package. A signed network was constructed using the blockwiseModules function in the WGCNA R package. A minimum module size of 30 genes was set. All other parameters were set to default. Following network construction, module eigengenes were calculated using the moduleEigengenes function in the WGCNA R package. The module-trait relationship was evaluated by determining the Pearson correlation between the module eigengene and the traits. Significance of each correlation was determined using the corPvalueFisher function in the WGCNA R package.

### Serum sample collection and RNA preparation

Venous blood samples were obtained from the 30 patients with AAAD when they were admitted to hospital and from the 30 volunteers at 07:00 am before breakfast. Serum samples were collected with pro-coagulation tubes, and centrifuged at 3000 rpm for 10 min at room temperature. Total RNA was extracted from each serum sample using Trizol LS Reagent (Life technologies, USA) according to the protocol from manufacturer. The tissue RNA extraction referred to the protocol in RNA preparation for RNA-Seq.

### Cells, plasmids construction and transfection

Primary human aortic smooth muscle cells (HASMCs) were purchased from ScienCell Research Laboratories (cat 6110). The circMARK3 cDNA was synthesized by Synbio Technologies (Suzhou, China). CircMARK3 cDNA was then cloned into the pCD-ciR vector (Geenseed biotech Co, Guangzhou, China). The already constructed circMARK3 cDNA-pCD-ciR vector was then electrotransfered (single pulse of square wave electroporation at 180V for 8ms, and 20 driving pulse cycles at 20v for 10ms) into the HASMCs to overexpress the circMARK3 using BEX CUY21 Electroporator (Japan).

### Quantitative reverse real-time PCR (qRT-PCR)

Total RNA (2μg) was treated by RNase-R for 15 min at 37°C using RNase-R (Epicenter) to remove linear transcripts. Treated RNA and total RNA (2μg) were reverse transcribed to synthesize cDNA using PrimeScript RT Master Mix (Perfect Real Time, TaKaRa), and total RNA (2μg) was reverse transcribed using the miRcute miRNA First-Strand cDNA Synthesis Kit (TIANGEN, China) according to the protocol from manufacturer. And then the cDNAs were analyzed by qRT-PCR with EvaGreen qPCR Mastermix-s (abm) and a LightCycler 480 II system (Roche). Beta-actin was used as the internal control for circRNA and mRNA, U6 was used as the internal control for miRs, and GAPDH was used as the internal control of serum circMARK3. The relative expression level of RNAs was calculated using the 2^-ΔΔCT^ method.

### Western blotting

Proteins from the aortic tissues were prepared with RIPA buffer (Cell Signaling Technology). Equal amounts of proteins were separated on SDS-PAGE, and electro-transferred to a PVDF membrane (Millipore). Antibodies that were used are as follow: anti-Fgr (1:1000, Abcam), anti-β-actin (1:1000, Abcam) and HRP-conjugated secondary antibody (1:5000, Jackson ImmunoR). The blots were detected by ECL western blotting substrate (Thermo Fisher). Images were captured and processed by Tanon5200Multi.

### Statistical analyses

Statistical analyses were performed using Prism 6 (GraphPad Software) and SPSS 22 (IBM). Data normality was checked using Kolmogorov-Smirnov (K-S) test to ensure that the statistical methods used in this study were appropriate. Results for the continuous variables are presented as Mean ± SD and for the categorical variables are presented as number and percentage. Differences between groups were assessed using Student’s *t* test or Wilcoxon’s rank-sum test for continuous variables and chi-square test or Fisher’s exact test for categorical variables, as appropriate. *P*<0.05 was considered statistically significant.

ROC curve was created to evaluate the diagnostic value of the circRNAs that were dysregulated in the serum of AAAD patients compared with those in controls. The area under curve (AUC) was used to assess the predictability of using serum circRNAs as biomarkers for AAAD. The definition of the Youden index is sensitivity plus specificity minus 1, and the highest Youden index corresponds to the optimal cut-off value. *P*< 0.05 was considered to represent statistical significance.

## Result

**Fig 1:**
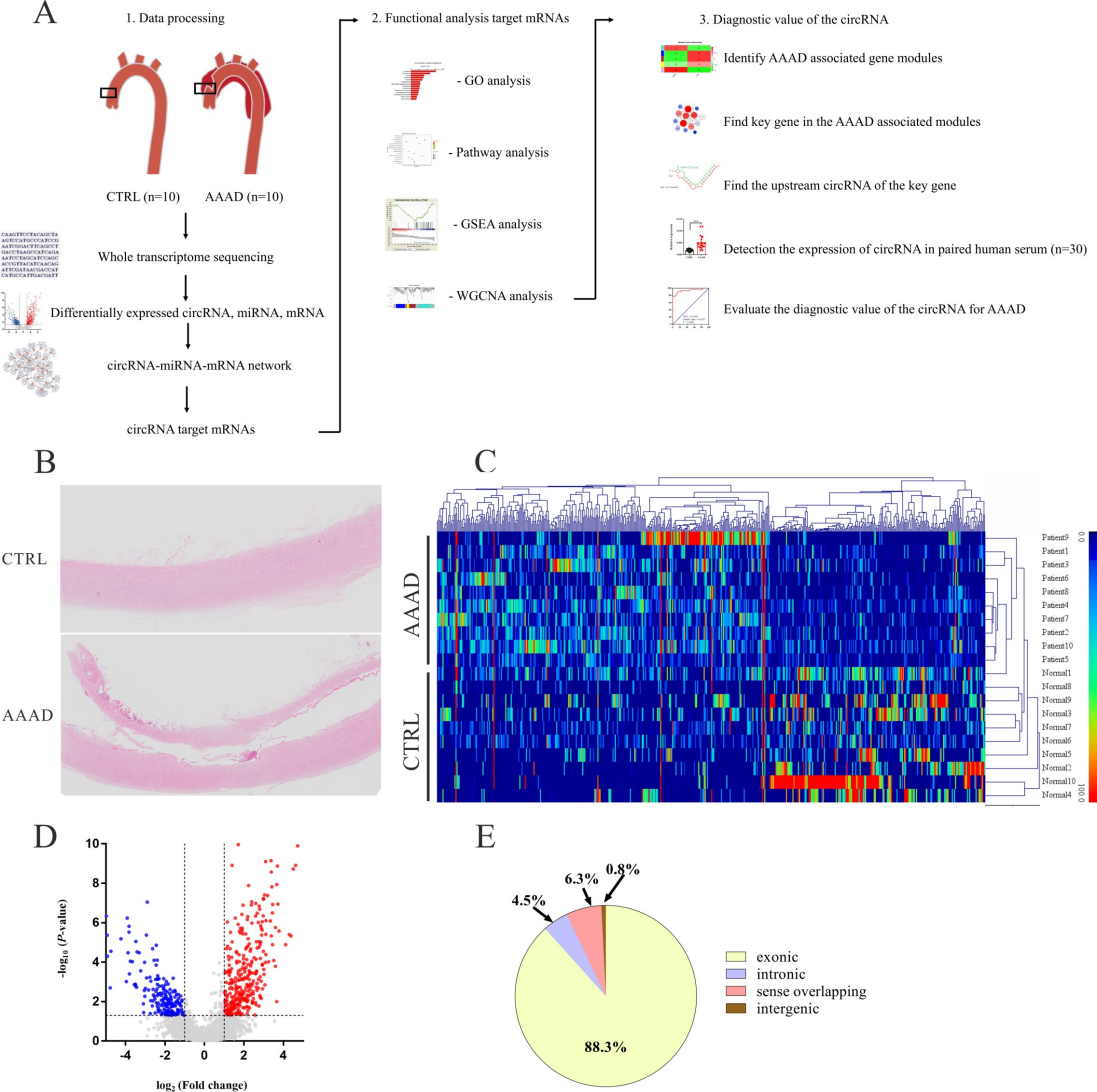
Differential expression of circRNAs between human acute Stanford type A aortic dissection (AAAD) tissues and normal aortic tissues. (A) The flowchart of this study. (B) Hematoxylin and eosin (HE)–stained sections showing the AAAD. (C) Hierarchical cluster analysis of all target circRNAs. 506 circRNAs were significantly differentially expressed (*P*<0.05, false discovery rate, FDR<0.05, fold change>2) between human AAAD tissues and normal aortic tissues. Among these, 320 circRNAs were upregulated and 186 circRNAs were downregulated in AAAD tissues. (D) Differentially expressed circRNAs were displayed by volcano plots. The blue and red parts indicated >2 fold decreased and -increased expression of the dysregulated circRNAs in AAAD tissues, respectively (*P*< 0.05). (E) Classification of differentially expressed circRNAs.

The experimental flow chart for this study is shown in Fig 1A and the histopathology typical of AAAD is shown in Fig 1B.

### Characteristics of patients

Age, gender, BMI, and other clinical characteristics of the patients in the discovery subset as well as those included in the full study cohorts are presented in Table 1.

**Table 1:** Clinical characteristics of patients who were enrolled in this study.

Characteristics	RNA-Seq Discovery Cohort			All enrolled patients		
	Control (n = 10)	AAAD (n = 10)	*P*	Control (n = 30)	AAAD (n = 30)	*P*
Age (years)	60.9±3.0	59.3±3.9	0.75	56.1±15.3	56.7±12.3	0.87
Male (%)	4 (40%)	5 (50%)	1.00	14 (47%)	19 (63%)	0.19
Height (cm)	163±2.4	168±1.5	0.07	164.8±7.0	168.1±6.7	0.06
Weight (kg)	63.3±3.0	71.3±4.5	0.16	66.6±8.6	69.6±13.0	0.24
BMI (kg/m^2^)	23.7±0.8	25.0±1.4	0.45	24.4±2.64	24.5±3.6	0.93
Hypertension (n)	3(30%)	7(70%)	0.18	20 (67%)	24(80%)	0.38
Diabetes mellitus (n)	0(0%)	1(10%)	1.00	4 (13.3%)	1(3.3%)	0.35
Smoking history (n)	0(0%)	0(0%)	1.00	0 (0%)	1 (3.3%)	1.00
Alcoholism (n)	0(0%)	0(0%)	1.00	6 (20%)	5 (16.7%)	1.00
CKD (n)	0(0%)	0(0%)	1.00	3 (10%)	2 (6.7%)	1.00
Stroke (n)	0(0%)	0(0%)	1.00	0 (0%)	1 (3.3%)	1.00

The *P* value refers to comparisons between the AAAD and the control group. The data are presented as the mean ± SD. BMI, body mass index; CKD, chronic kidney disease; AAAD, acute Stanford type A aortic dissection.

### Expression profiles of circRNAs

A total of 2935 circRNAs were detected by RNA-Seq, of which, 506 circRNAs were significantly dysregulated (*P*<0.05, FDR<0.05, fold change <0.5 or >2). 320 circRNAs were significantly up-regulated, and 186 circRNAs were significantly down-regulated in the AAAD group compared to controls. Hierarchical clustering and the volcano plot showed the expression profiles of circRNAs between AAAD and normal aortic tissues (Fig 1C and 1D). Among all the significantly dysregulated circRNAs, 88.3% of them were exonic type, 4.5% were intronic type, 6.3% were sense overlapping type, the left 0.8% were intergenic type (Fig 1E).

### qRT-PCR analysis of differentially expressed circRNAs, miRs and mRNAs

**Fig 2:**
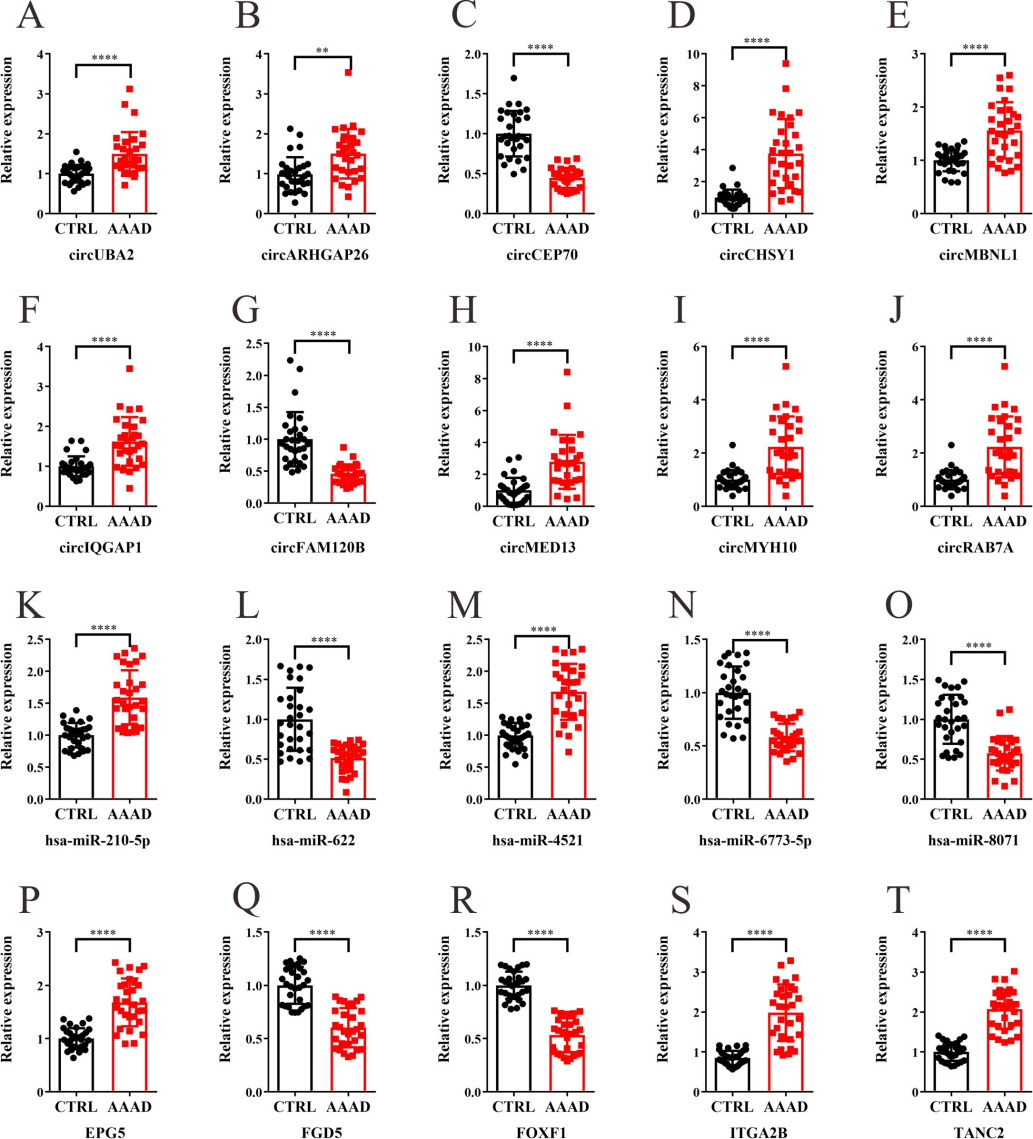
qRT-PCR analysis of differentially expressed circRNAs, miRs and mRNAs. The relative expression levels of the selected significantly differentially expressed circRNAs (A-J), miRs (K-O) and mRNAs (P-T) in 30 matched human AAAD and normal aortic tissues. The value of 2^-ΔΔCt^ was used to show the expression level of RNAs. Data are expressed as mean±SD. ****P < 0.0001.

The top 10 significantly differentially expressed circRNAs, 10 significantly differentially expressed miRs and 10 significantly differentially expressed mRNAs identified in the RNA-Seq analysis were selected for further qRT-PCR analysis. Thirty matched human AAAD and normal aortic tissues were used in this part, the results of qRT-PCR confirmed the up-regulation of circUBA2, circARHGAP26, circCHSY1, circMBNL1, circIQGAP1, circMED13, circMYH10, circRAB7A, hsa-miR-210-5p, hsa-miR-4521, hsa-miR-128-3p, hsa-miR-1180-5p, EPG5, ITGA2B, TANC2, FGR, IL-1α and TEAD2 and the down-regulation of circCEP70, circFAM120B, hsa-miR-622, hsa-miR-6773-5p, hsa-miR-8071, hsa-miR-139-3p, hsa-miR-187-3p, hsa-miR-1273-3p, FGD5, FOXF1,CDH5 and KCNK5 (Figs 2A–2T and S1).

### CircRNA-miRNA-mRNA interaction network

To further investigate the function of the differentially expressed circRNAs as miRNA sponges, differentially expressed circRNAs, miRs and mRNAs were analyzed together to construct circRNA-miRNA-mRNA interaction network. There were 678 significantly dysregulated target genes of differentially expressed circRNAs, among which 326 genes were significantly up-regulated, and 352 genes were significantly down-regulated in the AAAD group (S1 File).

### The Bioinformatics analysis of the target genes of the differentially expressed circRNAs

**Fig 3:**
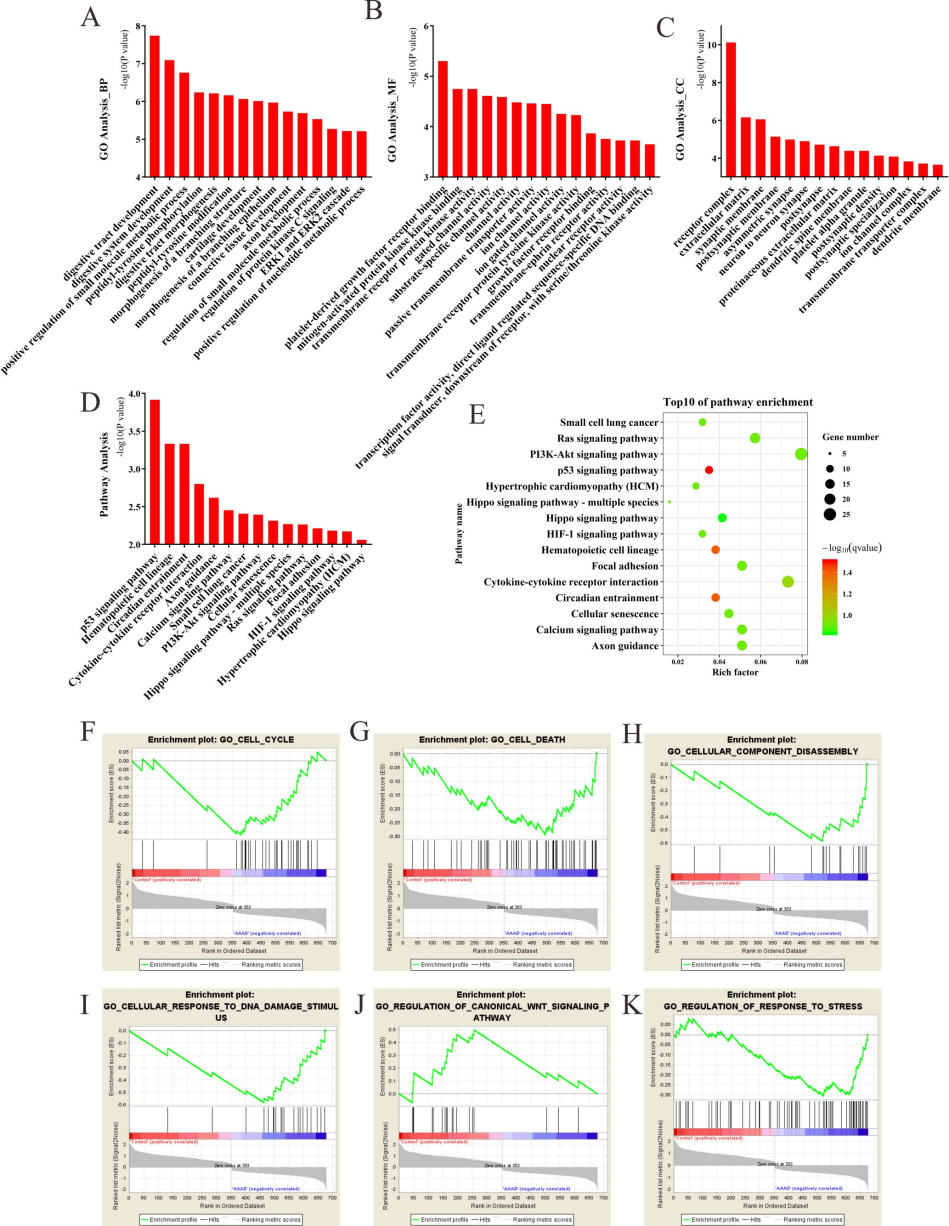
GO, KEGG pathway enrichment analysis and the gene set enrichment analysis (GSEA) of the target genes of differentially expressed circRNAs. (A) Biological process of GO analysis; (B) Cellular component of GO analysis; (C) Molecular function of GO analysis; (D) KEGG pathways analysis; (E) Scatterplot of enriched KEGG pathways. Y-axis represents pathway name and X-axis represents rich factor. Size and color of each bubble represents the number of differentially expressed genes enriched in the pathway and -log10(q-value), respectively. (F-K) GSEA enrichment plot. The GSEA enrichment plot for cell cycle (F), cell death (G), cellular component disassembly (H), response to DNA damage stimulus (I), regulation of canonical Wnt signaling pathway (J) and regulation of response to stress (K).

The most significantly enriched GO terms in the biological process, cellular component, and molecular function categories were digestive tract development, receptor complex and platelet-derived growth factor receptor binding, respectively (Fig 3A–3C). The results of KEGG pathway enrichment analysis showed that target genes of differentially expressed circRNAs were mainly involved in p53 signaling pathway, Ras signaling pathway, Calcium signaling pathway, as well as the cellular senescence (Fig 3D and 3E). The results of Gene set enrichment analysis (GSEA) revealed that the target genes were mainly enriched in cell cycle, cell death, cellular component disassembly, response to DNA damage stimulus, regulation of canonical Wnt signaling pathway and regulation of response to stress (Fig 3F–3K). These gene sets contained some known mechanisms that were previously reported in AAAD, such as cell death [24] and regulation of response to stress [7].

### Weighted gene co-expression network analysis (WGCNA) analysis of the target genes of differentially expressed circRNAs

**Fig 4:**
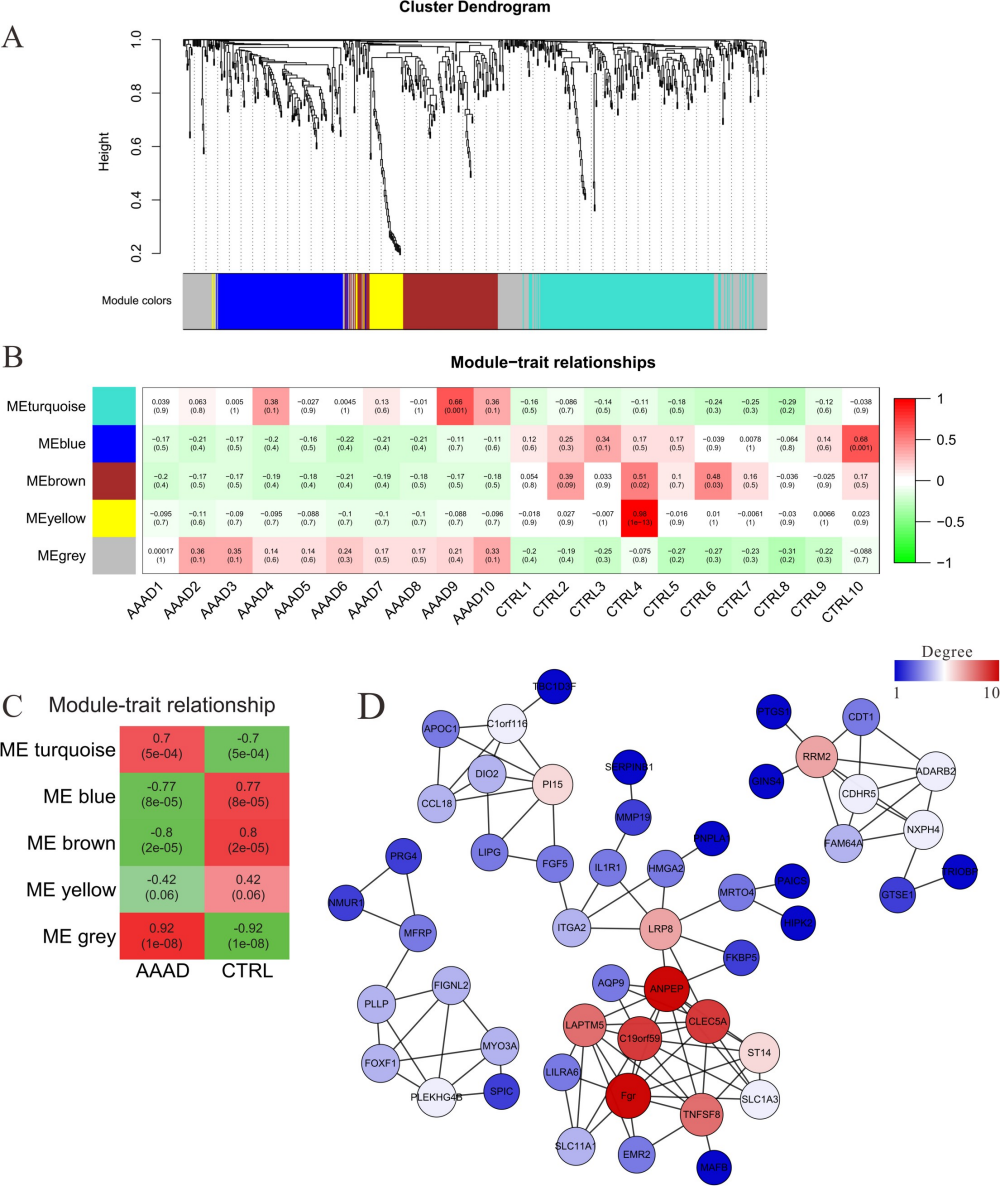
Weighted gene correlation network analysis (WGCNA) of the target genes of differentially expressed circRNAs. (A) The hierarchical cluster dendrogram of the target genes of differentially expressed circRNAs and the color assignments for each module. (B) The module-trait (sample) correlations and corresponding *P*-values. (C) The module-group correlations and corresponding *P*-values. (D) Gene co-expression network of the genes enriched in grey module.

**Fig 5:**
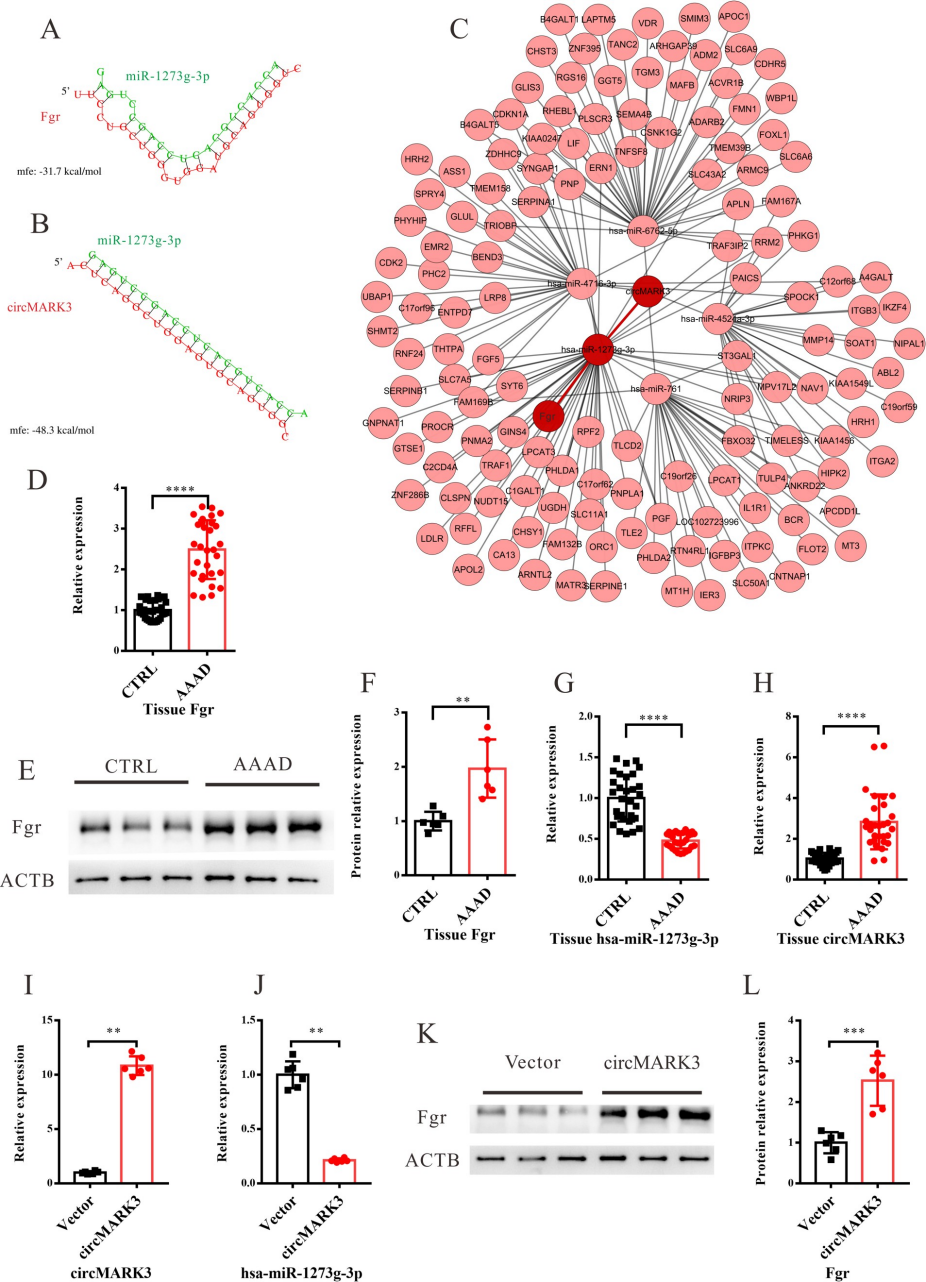
Prediction of circMARK3-miR-1273g-3p-Fgr interaction. (A) miR-1273g-3p targeting Fgr. (B) miR-1273g-3p targeting circMARK3. (C) Prediction of circMARK3-miRNA-mRNA interaction. The base-pairing and the minimum free energy (mfe) were predicted using the RNA hybrid program. (D-F) The relative expression levels of Fgr in the tissue of AAAD patients measured by qRT-PCR (D) and western blotting (E-F). (G-H) The relative expressions of miR-1273g-3p and circMARK3 in the tissue of AAAD patients. (I-L) HASMCs were transfected with circMARK3 or control vector. The relative expression of circMARK3 and hsa-miR-1273g-3p in HASMCs were measured by qRT-PCR assays (I-L). The expression of Fgr in the HASMCs were measured by western blotting assays (K-L). The value of 2^-ΔΔCt^ was used to show the expression level of RNAs. Data are expressed as mean±SD. **P < 0.01, ***P < 0.001 and ****P < 0.0001.

In this analysis, 678 differentially expressed genes were clustered into 5 modules (Figs 4A and S4–S7). The following module-trait relationship analysis revealed that the grey module was closely related to AAAD (Fig 4B and 4C). Gene co-expression networks were constructed for the genes contained in the grey module; the results showed that tyrosine-protein kinase Fgr was the core gene in the grey gene module (Fig 4D). The base-pairing and the minimum free energy (mfe) for the binding of Fgr to its targeting miRs and the mfe for the binding of miRs to its targeting circRNAs were predicted by the RNAhybrid program. Fgr-miR1273g-3p got the lowest mfe (-31.7 kcal/mol) among all Fgr-miRNA interaction pairs (Fig 5A). miR1273g-3p-circMARK3 got the lowest mfe (-48.3 kcal/mol) among all miR1273g-3p-circRNA interaction pairs (Fig 5B). These results predicted that the upstream circRNA of Fgr was circMARK3 (Fig 5A–5C). The expression of Fgr was significantly up-regulated in the tissues of AAAD, which was verified by qRT-PCR and Western blotting (Fig 5D–5F). The expression of miR1273g-3p was significantly down-regulated and circMARK3 was significantly up-regulated in tissues of AAAD (Fig 5G and 5H). To validate the interaction of circMARK3-miR1273-Fgr, circMARK3 was overexpressed in primary human aortic smooth muscle cells (HASMCs) (Fig 5I). The following qRT-PCR assays revealed that expression of hsa-miR-1273g-3p decreased significantly (Fig 5J), meanwhile, the western blotting assays showed that the expression of Fgr increased significantly in circMARK3 overexpression group (Fig 5K and 5L).

### ROC curve analysis of serum circMARK3 among patients

**Fig 6:**
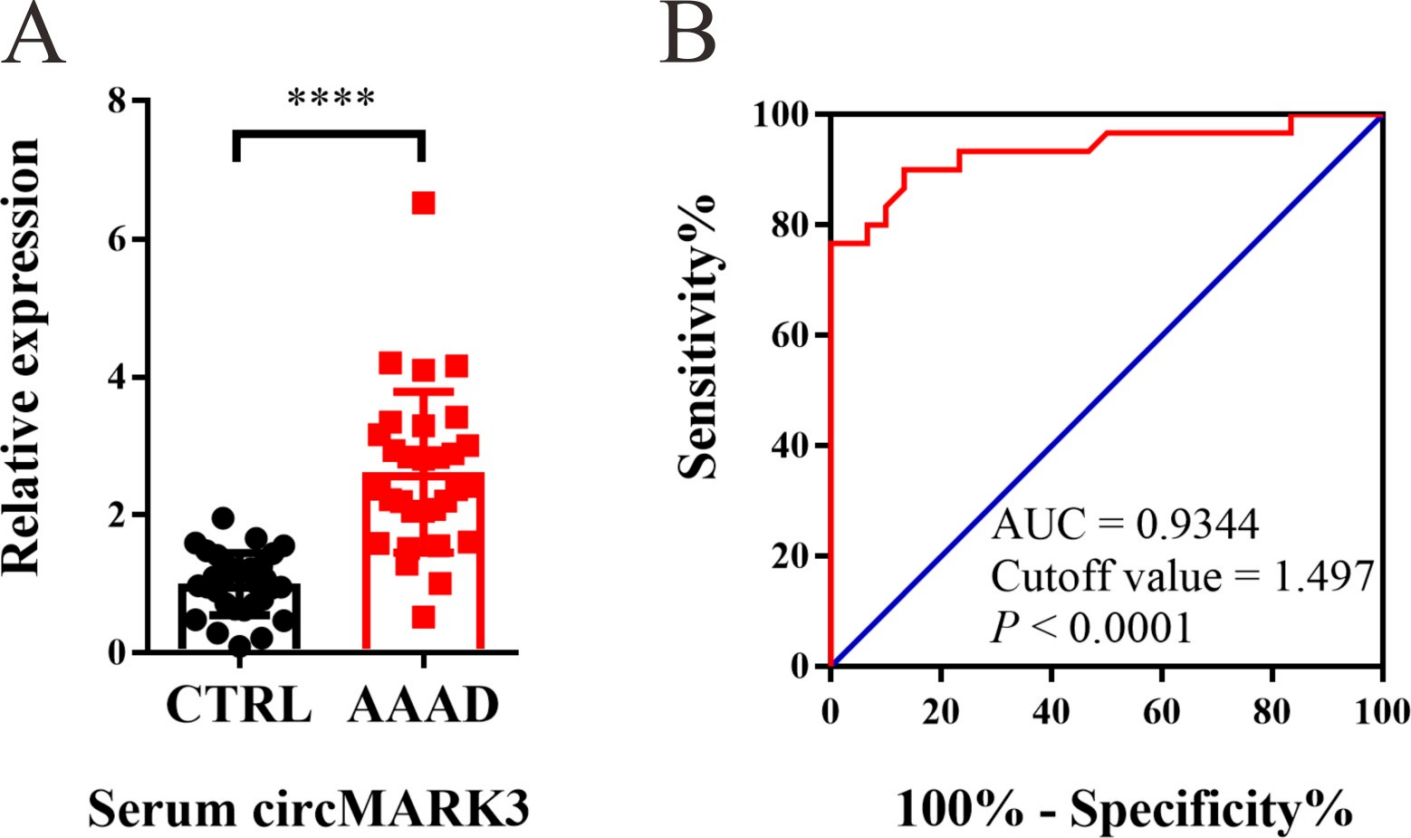
The diagnostic value analysis of serum circMARK3 for AAAD diagnosis. (A) circMARK3 was up-regulated in the serum of AAAD group. The value of 2^-ΔΔCt^ was used to show the expression level of RNAs. Data are expressed as mean±SD. ****P < 0.0001. (B) Receiver operating characteristic (ROC) curve showed the diagnostic value of serum circMARK3 for AAAD diagnosis (AUC = 0.9344, 95% CI: 0.8677–1.001, *P*< 0.0001).

circMARK3 expression was up-regulated in the serum of AAAD patients (Fig 6A). To evaluate the diagnostic value of serum circMARK3 in AAAD, a ROC curve was constructed, and the results showed that the AUC was 0.9344 (95% CI: 0.8677–1.001, *P*< 0.0001). The cutoff value was 1.497, and the corresponding sensitivity and specificity of serum circMARK3 were 90.0% and 86.7%, respectively (Fig 6B). Values below the cutoff value are negative, while those exceeding the cutoff value are positive. Therefore, circMARK3 may act as a potential biomarker for the diagnosis of AAAD.

MiRs are usually used as biomarkers in kinds of human disease [25], including aortic dissection [11]. We further evaluated the diagnostic value of the combination of serum circMARK3 and miR-1273-3p. The results revealed that the combination of circMARK3 and miR-1273-3p improved the predictability a bit. The AUC of the combination of circMARK3 and miR-1273-3p was 0.9644 (95% CI: 0.9238–1.005, *P*< 0.0001). The cutoff value was 0.4807, and the corresponding sensitivity and specificity was 93.3% and 86.7% respectively. (S2 Fig). The diagnostic values of serum circMARK3 as well as the combination of serum circMARK3 and miR-1273-3p were also verified in an independent patient cohort (S3 Fig).

## Discussion

Using the whole transcriptome RNA-Seq, we demonstrated that circRNAs were differentially expressed in human AAAD tissues compared with the control. One of the important functions of circRNAs is to serve as miRNA sponges to regulate the target genes of miRs [26]. Subsequently, circRNA-miRNA-mRNA network was constructed to find the target genes of differentially expressed circRNAs. We found Fgr, the target gene of circMARK3 was a core gene among the target genes of all differentially expressed circRNAs. In addition, circMARK3 could be a potential diagnostic biomarker for AAAD.

There is only previous one study about the differentially expressed circRNAs in human aortic dissection tissues (n = 3) using microarray, since the small sample size, the results could be uncertain [27]. A randomly chosen subset of the total AAAD cohort and propensity matched control samples were used to initially determine which, if any, circRNAs were differentially expressed with AAAD.

The top 2 terms enriched in the GO biological process are “digestive tract development” and “digestive system development”. Many of the enriched genes in the above biological processes were also proved to play important roles in the development of aortic related diseases, such as Cyclin-dependent kinase inhibitor 1 (CDKN1A), insulin-like growth factor II (IGF2), as well as myocardin (MYOCD). Previous studies reported that knockdown CDKN1A could rescue the aortic arch defect in foxe3 deficient zebrafish [8]. Zaina et al. demonstrated that IGF2 is a major promoter of growth of atherosclerotic lesions in apolipoprotein E defect mice, a widely used atherosclerosis animal model [28]. A large number of studies had revealed that MYOCD played vital roles in regulating the smooth muscle cells phenotype switching and was required for maintenance of aortic homeostasis [29, 30].

Pathway analysis and GSEA analysis showed that the target genes were mainly involved in p53 signaling pathway, cytokine-cytokine receptor interaction, calcium signaling pathway, cell death, cell cycle and Wnt signal transduction pathways. Apoptosis and necrosis of smooth muscle cells play an important role in AAAD [24]. And the related signaling pathways include Ras [31], PI3K- Akt [32] and p53 [33]. Previous studies had also shown that inhibiting Ras pathway can inhibit the aging of smooth muscle cells, thus reduce the incidence of aortic dissection [34]. Inhibiting p53 signal pathway could reduce the apoptosis of smooth muscle cells and aortic rupture caused by FOXE3 knockout [8]. In addition, smooth muscle phenotype transformation also plays an important role in the occurrence and development of aortic dissection [13, 35, 36], and the change of calcium signaling pathway is an important characteristic of smooth muscle cell phenotype transformation [37]. All the above results suggest that certain differentially expressed circRNAs might play important roles in AAAD.

WGCNA analysis was used to analyze the downstream target genes. The results provided evidence that Fgr was the core gene (own the highest K-core value) among the downstream target genes regulated by the differentially expressed circRNAs. Previous studies have showed that Fgr can transmit extracellular signals into cells, regulate immune response [20, 21] and releasing inflammatory factors, as well as facilitate cytoskeleton remodeling caused by extracellular stimulation [19]. The above signaling pathways regulated by Fgr also played an important role in the occurrence and development of AAAD [7], which suggests that Fgr might play an important role in the progress of AAAD. The WGCNA results also showed that Aminopeptidase N (ANPEP, owning the second highest K-core value) might play a role in the development of AAAD. Previous studies had reported that ANPEP, which is a member of rennin angiotensin system (RAS), could regulate the expression of angiotensin 2, 3, 4 [38, 39]. Moreover, RAS also had been implicated to play important roles in the development of aortic aneurysms and dissection [40, 41]. Thus, it is reasonable to suggest that ANPEP might also play vital roles in the development of AAAD. But further related biological experiment (both in vitro and in vivo) are still needed to elucidate the exact role of ANPEP in AAAD.

circMARK3 (the predicted upstream regulator of Fgr) was up-regulated in both serum and tissues of AAAD patients. Since circRNAs exhibit high stability, high conservation as well as aberrant expression in various human tissues, previous studies provide evidence that many of the circRNAs could be used as biomarkers for various human diseases [16, 42]. However, the diagnostic value of related circRNAs in AAAD has not been previously evaluated. Thus, in this study, RNA-Seq combined with the subsequent bioinformatics analyses were used to identify related circRNAs as a potential biomarker of AAAD. Therefore, circMARK3 was further analyzed as a potential biomarker. The diagnostic value of serum circMARK3 levels was determined from ROC analysis, the results of which demonstrate that circMARK3 was a sensitive and selective marker for late-stage AAAD. Considering that miRs and circRNAs are commonly used biomarkers for many kinds of human diseases [25], the diagnostic value of the combination of serum circMARK3 and miR-1273-3p was also evaluated. The combination of serum circMARK3 and miR-1273-3p further improved the diagnostic sensitivity and specificity, indicating that the combination of circRNAs and other biomarkers may provide better clinical utility.

### Limitations

This study had some limitations. First, the expression of circMARK3 in serum was shown to have potential diagnostic value in the final stage of aortic dissection, whether circMARK3 has predictive value in patients with risk factors of aortic dissection needs further investigation. Second, the expression of circMARK3 has not been evaluated in other cardiovascular diseases. Further studies evaluating the expression of circMARK3 in serum in a cohort of patients with other cardiovascular diseases are needed. Third, the other differentially expressed circRNAs could also act as the miRNA sponges, which inhibit miRs access to their target mRNAs by competing for the same binding site of miRs. However, their downstream targets and potential functions in the development of aortic dissection were only predicted by bioinformatic analysis, further biological experiments are needed to verify their exact roles in AAAD. Finally, although a reduction of miR-1273-3p was observed in HASMCs after circMARK3 overexpression, and the down-regulation of miR-1273-3p in AAAD specimens was accompanied with the up-regulation of Fgr. However, the relationship of circMARK3-miR1273-Fgr was only inferred, more biological experiments are still needed to verify the direct interactions among them.

In summary, this study provides a landscape of circRNAs expression profiles in AAAD and indicated that circMARK3 was a potential biomarker for the diagnosis of AAAD. Bioinformatic analysis predicts the potential functions of differentially expressed circRNAs. Further mechanistic and functional studies will aid in a deeper understanding of the pathological process of AAAD and find new specific molecular targets for clinical diagnosis and therapy.

## Supporting information

S1 Table Clinical characteristics of 1:1 matched patients and not-matched patients. (PDF)

S2 Table Clinical characteristics of 1:1 matched patients, 2:1 matched patients and total patients. (PDF)

S3 Table Sample information of RNA used in the RNA-Seq discovery cohort. (PDF)

S1 Fig qRT-PCR analysis of differentially expressed miRs and mRNAs. (TIF)

S2 Fig ROC curve showed the diagnostic value of the combination of serum circMARK3 and miR-1273-3p for AAAD diagnosis (AUC = 0.9644, 95% CI: 0.9238–1.005, P< 0.0001). (TIF)

S3 Fig ROC curve of serum circMARK3 as well as the combination of serum circMARK3 and miR-1273-3p for AAAD diagnosis in an independent cohort of patients. (A) ROC curve showed the diagnostic value of serum circMARK3 for AAAD diagnosis in an independent cohort of patients (AUC = 0.9211, 95% CI: 0.8499–0.9924, *P*< 0.0001). (B) ROC curve showed the diagnostic value of the combination of serum circMARK3 and miR-1273-3p for AAAD diagnosis in an independent cohort of patients (AUC = 0.9389, 95% CI: 0.8785–0.9993, *P*< 0.0001). n = 30 for each group.
(TIF)

S4 Fig Gene co-expression network of the genes enriched in blue module. (TIF)

S5 Fig Gene co-expression network of the genes enriched in brown module. (TIF)

S6 Fig Gene co-expression network of the genes enriched in turquoise module. (TIF)

S7 Fig Gene co-expression network of the genes enriched in yellow module. (TIF)

S1 File circRNA-miRNA-mRNA network. Differentially expressed circRNAs, miRs and mRNAs were analyzed together to construct circRNA-miRNA-mRNA interaction network.
(XLS)

S2 File Supplemental methods. (DOCX)
