# Supplementary material for: Expression profiles of circRNAs and the potential diagnostic value of serum circMARK3 in human acute Stanford type A aortic dissection
Source: PLoS One. 2019 Jun 28;14(6):e0219013. doi: 10.1371/journal.pone.0219013 (PMC6599129; doi:10.1371/journal.pone.0219013)
Supplement: S1 Table — (PDF) [file pone.0219013.s001.pdf]

**S1 Table. Clinical characteristics of 1:1 matched patients and not-matched patients**

| Characteristics | 1:1 matched | | | Not-matched | | |
|---|---|---|---|---|---|---|
| | Control (n=30) | AAAD (n=30) | *P* | Control (n=142) | AAAD (n=29) | *P* |
| **Age (years)** | 56.1±15.3 | 56.7±12.3 | 0.87 | 63.7±9.9 | 47.1±14.1 | <0.0001 |
| **Male (%)** | 14 (47%) | 19 (63%) | 0.19 | 96 | 24 | 0.12 |
| **Height (cm)** | 164.8±7.0 | 168.1±6.7 | 0.06 | 166.3±8.3 | 168.1±7.7 | 0.28 |
| **Weight (kg)** | 66.6±8.6 | 69.6±13.0 | 0.24 | 65.3±10.9 | 82.2±19.8 | <0.0001 |
| **BMI (kg/m$^2$)** | 24.4±2.64 | 24.5±3.6 | 0.93 | 24.2±3.2 | 27.1±5.6 | 0.0002 |
| **Hypertension (n)** | 20 (67%) | 24(80%) | 0.38 | 69 | 26 | <0.0001 |
| **Diabetes mellitus (n)** | 4 (13.3%) | 1(3.3%) | 0.35 | 32(22.5%) | 0 (0%) | 0.0028 |
| **Smoking history (n)** | 0 (0%) | 1 (3.3%) | 1.00 | 35 | 8 | 0.81 |
| **Alcoholism (n)** | 6 (20%) | 5 (16.7%) | 1.00 | 15 | 7 | 0.07 |
| **CKD (n)** | 3 (10%) | 2 (6.7%) | 1.00 | 2 | 0 | 1.00 |
| **Stroke (n)** | 0 (0%) | 1 (3.3%) | 1.00 | 0 | 1 | 0.17 |
