# Supplementary material for: Expression profiles of circRNAs and the potential diagnostic value of serum circMARK3 in human acute Stanford type A aortic dissection
Source: PLoS One. 2019 Jun 28;14(6):e0219013. doi: 10.1371/journal.pone.0219013 (PMC6599129; doi:10.1371/journal.pone.0219013)
Supplement: S2 Table — (PDF) [file pone.0219013.s002.pdf]

S2 Table. Clinical characteristics of 1:1 matched patients, 2:1 matched patients and total patients

Characteristics	1:1 matched			2:1 matched			Total		
	Control (n=30)	AAAD (n=30)	*P*	Control (n=47)	AAAD (n=30)	*P*	Control (n=172)	AAAD (n=59)	*P*
**Age (years)**	56.1±15.3	56.7±12.3	0.87	57.8±13.2	56.7±12.3	0.71	62.4±11.1	52.0±13.2	<0.0001
**Male (%)**	14 (47%)	19 (63%)	0.19	27(57%)	19 (63%)	0.64	110(64%)	43 (73%)	0.26
**Height (cm)**	164.8±7.0	168.1±6.7	0.06	165.1±7.2	168.1±6.7	0.07	166.1±8.1	168.1±7.2	0.09
**Weight (kg)**	66.6±8.6	69.6±13.0	0.24	66.7±9.1	69.6±13.0	0.25	65.5±10.5	74.4±16.7	<0.0001
**BMI (kg/m^2)**	24.4±2.64	24.5±3.6	0.93	24.7±3.2	24.5±3.6	0.80	24.2±3.1	25.8±4.7	0.0033
**Hypertension (n)**	20 (67%)	24(80%)	0.38	32(68%)	24(80%)	0.30	89(52%)	50(85%)	0.23
**Diabetes mellitus (n)**	4 (13.3%)	1(3.3%)	0.35	4(8%)	1(3.3%)	0.64	36(21%)	1(1.7%)	0.0001
**Smoking history (n)**	0 (0%)	1 (3.3%)	1.00	2(4.3%)	1 (3.3%)	1.00	35(20%)	9(15%)	0.447
**Alcoholism (n)**	6 (20%)	5 (16.7%)	1.00	8(17%)	5 (16.7%)	1.00	21(20%)	12(20%)	0.133
**CKD (n)**	3 (10%)	2 (6.7%)	1.00	4(8.5%)	2 (6.7%)	1.00	5(2.9%)	2(3.4%)	1.00
**Stroke (n)**	0 (0%)	1 (3.3%)	1.00	0(0%)	1 (3.3%)	0.39	0(0%)	2(3.4%)	0.06
