# Supplementary material for: Expression profiles of circRNAs and the potential diagnostic value of serum circMARK3 in human acute Stanford type A aortic dissection
Source: PLoS One. 2019 Jun 28;14(6):e0219013. doi: 10.1371/journal.pone.0219013 (PMC6599129; doi:10.1371/journal.pone.0219013)
Supplement: S3 Table — (PDF) [file pone.0219013.s003.pdf]

**S3 Table. Sample information of RNA used in the RNA-Seq cohort.**

## Sample Info

| Well | RIN$^e$ | Vol. [ul] | Conc. [ng/ul] | Sample Description |
|---|---|---|---|---|
| A0 | | | | |
| A1 | 8.8 | 40 | 293 | CTRL1 |
| B1 | 8.5 | 40 | 265 | CTRL2 |
| C1 | 8.4 | 40 | 290 | CTRL3 |
| D1 | 8.6 | 40 | 277 | CTRL4 |
| E1 | 8.7 | 40 | 269 | CTRL5 |
| F1 | 8.3 | 40 | 267 | CTRL6 |
| G1 | 8.5 | 40 | 269 | CTRL7 |
| H1 | 8.6 | 40 | 262 | CTRL8 |
| A2 | 8.9 | 40 | 281 | CTRL9 |
| B2 | 8.4 | 40 | 279 | CTRL10 |
| C2 | 8.5 | 40 | 256 | AAAD1 |
| D2 | 8.7 | 40 | 273 | AAAD2 |
| E2 | 8.8 | 40 | 298 | AAAD3 |
| F2 | 8.6 | 40 | 257 | AAAD4 |
| G2 | 8.3 | 40 | 271 | AAAD5 |
| H2 | 8.5 | 40 | 286 | AAAD6 |
| A3 | 8.9 | 40 | 287 | AAAD7 |
| B3 | 8.7 | 40 | 285 | AAAD8 |
| D3 | 8.4 | 40 | 281 | AAAD9 |
| E3 | 8.5 | 40 | 257 | AAAD10 |
