# Supplementary figures and images for: Expression profiles of circRNAs and the potential diagnostic value of serum circMARK3 in human acute Stanford type A aortic dissection
Source: PLoS One. 2019 Jun 28;14(6):e0219013. doi: 10.1371/journal.pone.0219013 (PMC6599129; doi:10.1371/journal.pone.0219013)

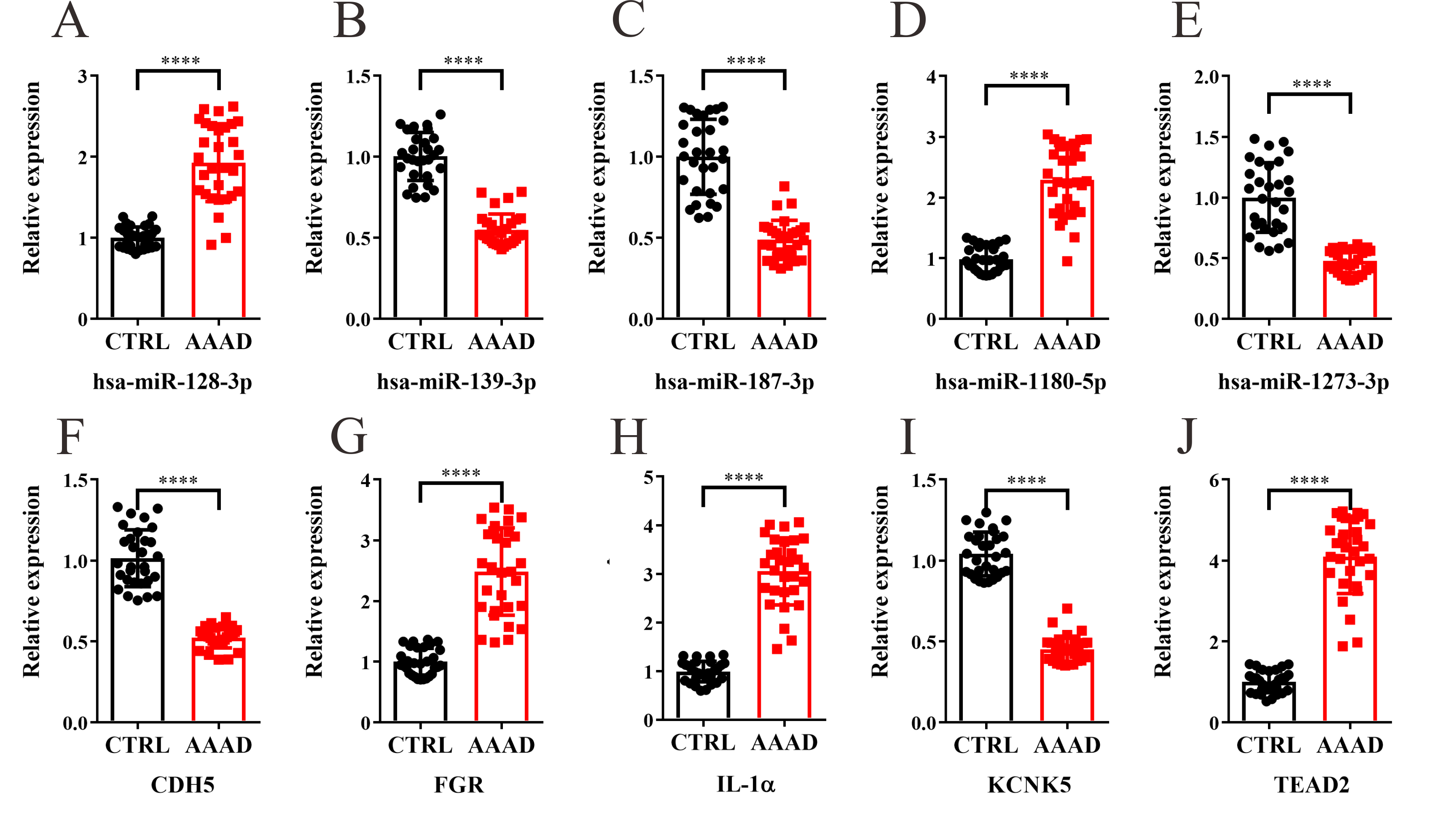

Supplement: S1 Fig — (TIF) [file pone.0219013.s004.TIF]

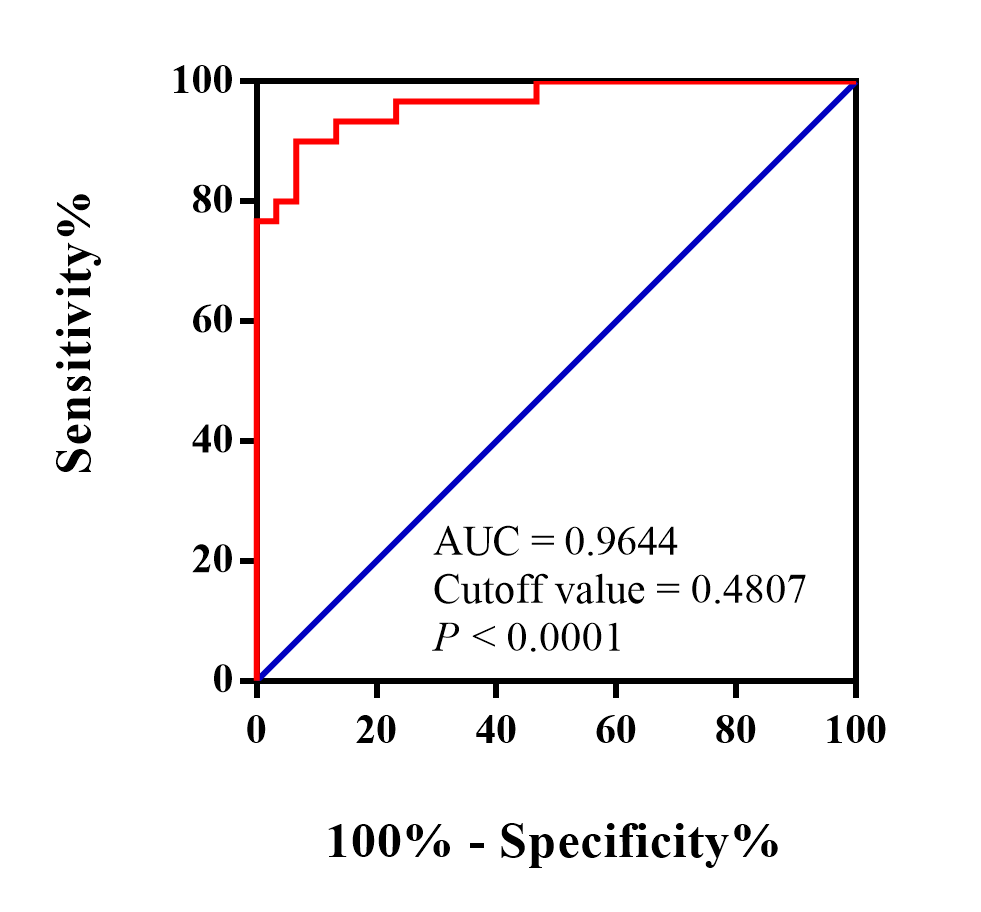

Supplement: S2 Fig — (TIF) [file pone.0219013.s005.TIF]

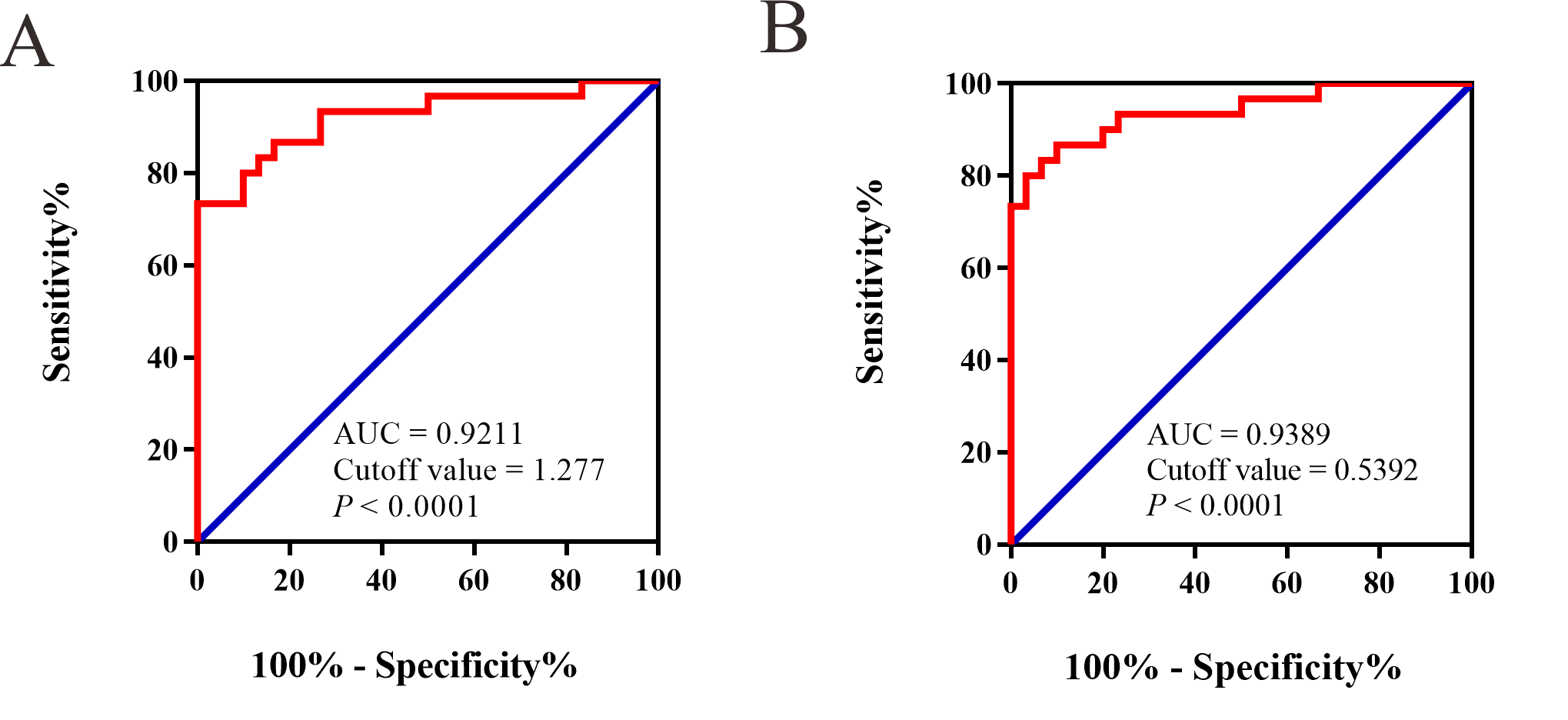

Supplement: S3 Fig — (A) ROC curve showed the diagnostic value of serum circMARK3 for AAAD diagnosis in an independent cohort of patients (AUC = 0.9211, 95% CI: 0.8499–0.9924, P< 0.0001). (B) ROC curve showed the diagnostic value of the combination of serum circMARK3 and miR-1273-3p for AAAD diagnosis in an independent cohort of patients (AUC = 0.9389, 95% CI: 0.8785–0.9993, P< 0.0001). n = 30 for each group. (TIF) [file pone.0219013.s006.TIF]
