# Supplementary material for: Expression profiles of circRNAs and the potential diagnostic value of serum circMARK3 in human acute Stanford type A aortic dissection
Source: PLoS One. 2019 Jun 28;14(6):e0219013. doi: 10.1371/journal.pone.0219013 (PMC6599129; doi:10.1371/journal.pone.0219013)
Supplement: S2 File — (DOCX) [file pone.0219013.s012.docx]

**Supplemental Methods**

**ROC curve for the combination of serum circMARK3 and miR-1273-3p**

To evaluate the diagnostic value of the combination of serum circMARK3 and miR-1273-3p, binary logistic regression was used to calculate the predicted value by SPSS 22. The covariates were the expression levels of serum miR-1273-3p and serum circMARK3. The predicted value was used to construct the ROC curve.
